# Factors associated with injuries among preschool children in Egypt: demographic and health survey results, 2014

**DOI:** 10.1186/s12889-020-08658-w

**Published:** 2020-05-01

**Authors:** Zeinab Mohammed, Ali Aledhaim, Eman Mohammed AbdelSalam, Maged El-Setouhy, Mohamed EL-Shinawi, Jon Mark Hirshon

**Affiliations:** 1 Public Health and Community Medicine Department, Faculty of Medicine, Beni-Suef University, Beni Suef, Egypt; 2 Department of Emergency Medicine, University of Maryland School of Medicine, Baltimore, MD USA; 3 Department of General Surgery, Faculty of Medicine, Beni-Suef University, Beni Suef, Egypt; 4 Department of Family and Community Medicine, Faculty of Medicine, Jazan University, Jazan, Saudi Arabia; 5 Department of Community, Environmental and Occupational Medicine, Faculty of Medicine, Ain Shams University, Cairo, Egypt; 6 Department of General Surgery, Ain Shams University, Cairo, Egypt; 7 Department of Epidemiology and Public Health, University of Maryland School of Medicine, Baltimore, MD USA

**Keywords:** Preschool children, Injuries, Demographic and health survey, Egypt

## Abstract

**Background:**

Childhood injuries are a significant and growing global public health problem, often with high morbidity and, at times, mortality. A large proportion of injuries in preschool children occur in or around the home. We aimed to identify socioeconomic and demographic factors associated with preschool children injuries in Egypt.

**Methods:**

Secondary data analysis were done for the Egyptian Demographic and Health Surveys (EDHS), 2014. Potential associated factors were measured from data on child welfare and questions on the prevalence of accidents and injuries of preschool children. These data were linked to the children demographic data, maternal age at marriage, working status of the mother, and questions on childcare arrangements.

**Results:**

Out of the 634 injured children, 520 (83.4%) children required medical care for their injuries. The most common reported injury was an open wound 288 (45.5%), followed by fractures 237 (35.7%), burns 124 (19.7%), electrical shock 12 (1.9%) and other unknown types of injury 15 (2.4%). There was a positive correlation between injury and child’s age, household wealth, mother’s age at marriage, and unsupervised children or children left in the care of a minor.

**Conclusion:**

Leaving children unsupervised or in the presence of other young children is significantly associated with the occurrence of child injuries.

## Background

Unintentional injuries are major causes of mortality and morbidity in children, resulting in over 875,000 deaths annually worldwide [1]. According to the World Health Organization (WHO), one million children die every year due to injury and violence related causes [2]. Globally, approximately 95% of children who sustain injuries live in low- or middle-income countries (LMICs) [3]. Injuries are the leading cause of death among 2 to 4 years old children. Moreover, millions of children require medical care, including hospitalization due to nonfatal injuries [4]. Nearly, half of all preschool childhood injuries occurring at home [5]. Preschool children (0–4 years) are more vulnerable to home injuries because they spend most of their time indoors [6].

The nature of childhood injuries occurring in the household has been well described in developed countries, but they are less well understood in LMICs in which most of the global morbidity and mortality occur [7]. Egypt, as a Middle Eastern LMIC, has substantial burden from injuries. In Egypt, a household survey conducted in 2011 demonstrated that childhood injuries accounted for a large burden on the healthcare system as 95% of the surveyed children had at least one injury [8]. A WHO study of children conducted in four different developing countries concluded that Egypt had the highest prevalence (32%) of childhood injuries [9]. In LMICs like Egypt, challenging living conditions, including poor housing infrastructure, lack of protective barriers to cooking or washing areas, and inadequate recreational space, are among the hazards that place young children at risk for burns, poisoning, and falls [10]. Additionally, access to appropriate injury medical care is frequently more challenging, especially for poor or rural families. Many injured children receive care primarily from their families [11]. Maternal age, maternal education, and working status have been also associated with an increased likelihood of childhood injuries for children up to 18 years of age [8].

Despite the substantial burden, there is a lack of knowledge concerning factors associated with injuries among preschool children in Egypt. The objective of this study is to determine the socioeconomic and demographic factors associated with injuries for preschool children in Egypt.

## Methods

### Study design

We conducted a secondary data analysis of a population-based cross-sectional study based on the 2014 data from the Egyptian Demographic and Health Survey (EDHS) [12]. The Demographic and Health Survey (DHS) program collects a substantial amount of information on different topics such as family planning, domestic violence, nutrition, anemia prevalence, and child and maternal health for a sample of participating international countries. These databases are publicly available to researchers and scientists through the DHS website. The information is processed and presented in reports and data formats that describe the situation of the relevant country. The 2014 Egyptian EDHS was the last standard survey conducted and was the first national health survey in Egypt since 2008. The information is processed and presented in reports and data formats that describe the situation of the relevant country. The 2014 Egyptian EDHS was the last standard survey conducted and was the first national health survey in Egypt since 2008 [12].

### Sampling techniques

Detail information about the EDHS sampling technique are published in the EDHS 2014 final report [12]. The EDHS involved a multi-stage sampling design. Administratively, Egypt is divided into 27 governorates (Additional file 1). Each governorate is further sub-divided into towns and villages. All *shiakhas* (urban administrative units) and villages in Egypt constituted the sampling frame for the 2014 EDHS. The list of administrative units was prepared by the Egyptian Central Agency for Public Mobilization and Statistics (CAPMAS).

### Study population

#### Data management

The 2014 EDHS included specific questions relating to injuries or accidents of preschool children (Additional file 2). Two questionnaires were used in data collection via a household interview. The household questionnaire was administered to the head of the household; it collected information on potential risk factors including childhood indicators (age, sex, and residence) and household indicators (wealth index, and reported child care level). The individual questionnaire was administered to all ever-married women aged 15–49 who were usual members of the selected households; it collected data about marital status, educational attainment, mothers’ age at the time of marriage, and maternal working status.

The design weights were further adjusted for household non-response as well as for individual non-response to get the sampling weights for households.

### Primary outcome & measures

The primary outcome was child injury defined as ever being injured or involved in an accident at home. Three questions related to this outcome were asked by the DHS survey. The first was whether the child was ever injured or involved in an accident at home, the second one was about the type of injury (ies) or accident(s) and the third was whether the injury or accident require medical care.

Associated factors examined included: child age, gender, the area of residence, mother education, wealth index, mother age at marriage and child supervision. Child age was divided into two groups; less than 2 years and 2 to 4 years old. Educational level attained by mothers were categorized into three groups, no education at all, some education (which meant that the mother gained incomplete or complete primary education), and the last group for mothers that had gained secondary or higher education. The financial status in the EDHS was initially divided into five groups based on a set of data collected to measure the wealth quintiles (lowest, second, middle, fourth, and highest) which were condensed to three categories (low, middle and high) for analysis by combining the first two groups together (lowest and second) and the last two groups (fourth and highest) together. Child supervision was based upon two variables: left alone or in the care of a minor during the preceding week for more than 1 h.

### Sampling weights

The DHS uses a cluster multistage sampling design to collect survey data. Weights are applied to account for non-respondent and unequal selection probability. Hence, our findings were weighted and adjusted accordingly. A complete description of weights and their use has been published elsewhere (DHS Methodology report reference).

### Statistical analysis

A survey design was set up prior to data analysis to account for the EDHS sampling approach. Descriptive statistics were presented as frequencies and percentages. Bivariate analyses were used to examine associations between the explanatory variables and childhood injury using Chi-square tests and Fisher correction tests if needed. Multivariable logistic regression was utilized to estimate the effect of each associated factors. All tests were two-sided and a *p*-value < 0.05 was deemed statistically significant. Analyses were conducted using SPSS version 22.

## Results

The EDHS surveyed 29,471 households, of which 1296 households refused to participate and 28,175 households were successfully interviewed. Households with preschool children accounted for 14,856, all of whom were included in the analysis. In the sample, 634 (4.3%) of the children were injured or involved in accidents at home (Table 1). The most common injury was opened wounds, *n* = 288 (45.5%), followed by fractures, *n* = 237 (35.7%), and burns, *n* = 124 (19.7%). Medical care was provided for 83.5% of all injured children (Table 1).

**Table 1:** Injury Description of Preschool Children, EDHS 2014

*Data Element*	*Frequency*	*Percentage*
**Injury at Home**		
No	14,216	95.7
Yes	634	4.3
**Injury Type**		
Burn	110	17.3
Fracture	209	32.9
Open wound	288	45.5
Electric	12	1.8
Other	15	2.4
**Requiring Medical Care**		
No	103	16.5
Yes	520	83.5

Characteristics associated with injuries in preschool children are presented in Table 2. Older children (2–4 yrs.) had a higher prevalence of injuries (6.1%) compared to children less than 2 years of age (1.8%). Male children had a higher prevalence of injuries than female children (5% versus 3.5%, respectively). Lower wealth index households and younger maternal marriage age had higher injury proportions compared to higher wealth index and older marriage age. Injuries occurring when a child was left alone or with the care of a minor accounted for 13.6 and 15.2%, respectively.

**Table 2:** Factors Associated with Injuries in Preschool Children, EDHS 2014

Studied variables	Child injuries		Total	***P*** value
	No = 14,216 N (%)	Yes = 633 N (%)		
**Sociodemographics**				
**Age**				< 0.001*
<2 years	6177 (98.2)	111 (1.8)	6288	
2–4 years	8039 (93.9)	522 (6.1)	8561	
**Gender**				< 0.001*
Male	7422 (95.0)	390 (5.0)	7812	
Female	6794 (96.5)	243 (3.5%)	7037	
**Urban Rural Residence**				0.001*
Urban	4423 (96.5)	159 (3.5)	4582	
Rural	9794 (95.4)	474 (4.6)	10,268	
**Place of Residence**				
Urban	1437 (96.2)	56 (3.8)	1493	0.87
Governorates	1297 (95.7)	58 (4.3%)	1355	0.94
Lower Urban	5414 (94.4%)	319 (5.6%)	5733	< 0.001*
Lower Rural	1607 (97.5%)	41 (2.5%)	1648	0.001*
Upper Urban	4313 (96.5%)	155 (3.5%)	4468	0.01*
Upper Rural Frontiers	148 (97.4%)	4 (2.6%)	152	0.04*
**Mothers’ education**				
No education	2569 (96.5)	92 (3.5)	2661	0.09
Some education	3194 (95.3)	159 (4.7)	3353	0.13
Secondary/higher education	8273 (95.8)	363 (4.2)	8636	0.96
**Wealth index**				
Low	5226 (95.0)	274 (5.0)	5500	0.01*
Middle	3602 (95.9)	155 (4.1)	3757	0.69
High	5388 (96.3)	205 (3.7)	5593	0.03*
**Maternal Factors**				
**Marriage age (yrs)**				
9–19	6634 (95.2)	338 (4.8)	6972	0.01*
20–28	6835 (96.2)	272 (3.8)	5832	0.12
29–44	414 (98.4)	7 (1.6)	421.1	0.16
**Mother working status**				
Yes	1772 (95.9)	75 (4.1)	1847	0.64
No	12,091 (95.7)	543 (4.3)	12,634	
**Care Level**				
**Left alone (> 1 h)**				
Yes	405 (86.4)	64 (13.6)	469	< 0.001*
No	13,811 (96.)	569 (4.0)	14,380	
**Left with a Minor (< 10 yr)**				
Yes	340 (84.8)	61 (15.2)	401	< 0.001*
No	13,876 (96.0)	572 (4.0)	14,448	

*Significance *p* < 0.05

The weighted adjusted odds ratios of contributory factors associated with preschool childhood injuries are listed in Table 3. Older children (2–4 yrs.) were three times more likely to be injured compared to younger children (95% CI, 2.6–4.5), controlling for gender, place of residence, wealth level, mother marriage age, left alone and left alone with a minor. A male child had approximately 50% more chance of being injured than a female child (95% CI, 1.2–1.8). Low-income households had a 1.38 injury odds ratio compared to their middle-income counterparts. Also, children of younger mothers (< 20 yrs.) were 3 times more likely to get injured compared to relatively older mothers (> 29 yrs), (95% CI, 1.1–8.7). Children who were left alone at home or in the care of a minor had over twice the odds of being injured relative to those not left alone or in the care of an adult.

**Table 3:** Weighted Odds Ratios for Contributory Factors and Injury in Preschool Children

The studied Factor	Multivariate logistic regression			
	OR	[95% CI]		*P*-value
Age				
2–4 years	3.45	2.64	4.50	< 0.001
< 2 years	1			
Gender				
Female	1			< 0.001
Male	1.47	1.20	1.82	
Place of Residence				
Upper Urban	1			
Frontier Gov	0.95	0.45	1.98	0.89
Urban gov	1.63	1.01	2.62	0.04
Lower Urban	1.73	1.06	2.80	0.03
Lower Rural	2.08	1.34	3.24	0.001
Upper Rural	1.17	0.72	1.91	0.652
Wealth level				
Middle	1			
Low	1.38	1.04	1.84	0.03
High	1.13	0.78	1.66	0.51
Marriage Age				
29-44 yr	1			
9-19 yr	3.07	1.08	8.72	0.04
20-28 yr	2.39	0.84	6.83	0.10
Left alone (> 1 h)				
No	1			
Yes	2.08	1.30	3.35	0.002
Left with a Minor (< 10 yr)				
No	1			
Yes	2.63	1.65	4.18	< 0.001

## Discussion

This Egypt wide study showed positive correlations between preschool childhood injury and child age, household wealth, mother marriage age, and left alone children or left in the care of a minor. These findings are consistent with published literature. A study conducted on 924 hospitalized children in China found that 0–3 years are the most vulnerable age for injuries [13]. Another study concluded that injuries were the leading cause of death between 2009 and 2014 in Hunan province in China [14]. A community-based survey over 27 governorates in Egypt revealed that injury is significantly higher in children 2–6 years compared to younger and older children [8]. Child mortality due to injury shows a sustained high prevalence in developing countries rather developed countries [15].

Socioeconomic status is a historical risk factor of childhood injuries. A number of publications showed that it is negatively associated with children injuries [16–18]. Our study found children of low-income households to have a 38% more chance of being injured compared to middle or high-income households. A study in Bangladesh revealed that burn injuries in preschool children were associated with maternal illiteracy and low socioeconomic level [18]. Another study in England revealed that the incidence of thermal injuries was higher among children of low socioeconomic level [19].

The highest prevalence of injuries was seen when the child was left alone (13.6%) or with a less than 10 years old minor (15.2%). Children of working mothers had more than double the odds of being alone or with a minor compared to non-working mothers. While we could not discern the level of supervision, many studies discussed the importance of caregiver supervision and child injuries [20, 21]. Others indicated that high care level was associated with decreased risk of injury. Davis, et al., showed that supervision has a role in decreasing dog bites [22]. Schwebel, et al., highlighted that increasing the quality of supervision reduced the risk of unintentional injury and proximal maternal supervision was important to prevent injuries in a low-income setting [23]. Therefore, preschoolers are more liable to sustain injuries when they are not under proper adult supervision.

In the sample, 47% of mothers were married before reaching their 20th birthday, which is relatively uncommon in Middle Eastern culture, but common in some rural and low socioeconomic areas. Children of these mothers were three times more likely to sustain an injury compared to mothers married at a relatively older age (> 29 yrs.). Damashek and Corlis [24] showed that young maternal age associated with children hospitalization due to injury. Another study in Sweden [25] showed that children of the teenage mother were at potential risk for injuries and that injury prevention programs were warranted.

### Limitations

While this large sample population-based study was designed to be representative of the population in Egypt, it still has limitations. First, the analysis is in retrospect and the survey was not specifically designed to collect all factors contributing to preschool childhood injuries. The constant presence of other preschool relatives could be a contributing risk leading to horseplay and injury. In addition, the injury-related data lacks certain granularity. Although the type of injury and medical care status is known, injury acuity and the level of care given is unknown. Second, the data is prone to two types of bias: recall bias and reporting bias. Recall bias is intrinsic in the fact that recent or severe injuries are more likely to be remembered. Reporting bias is possible since survey respondents were not necessarily child mothers. Hence, a survey respondent may not be the most knowledgeable person about the injury history of a child or any other related information. Therefore, the DHS cautions about literal data interpretations.

## Conclusion

Our Egypt wide analysis showed that factors contributing to preschool injuries were child age, gender, mother’s marriage age, socioeconomic status, being left alone, and left in a minor’s care. Of particular note are issues related to adequate supervision of young children. Protecting children’s safety and preventing injuries is an essential job of the caregiver. They need to be attentive and provide close supervision at all times, both outside and inside, and teach children how to keep themselves safe. Preventing injuries is challenging but it may be lifesaving for many children. Further granular investigations are needed to assess the need for support programs to low-income households and prevention education to mothers marrying at a younger age.

## Supplementary information

**Additional file 1.** Demographic variables: Place of residency.

**Additional file 2.** The questions used to assess if the child was involved in an injury or an accident: 034 Has (NAME) ever been injured or involved in an accident at home? YES, NO.

## Data Availability

The datasets generated for the current study are available from the corresponding author on reasonable request. The dataset supporting the conclusions of this article is available in the Measure DHS repository. https://www.dhsprogram.com/data/dataset/Egypt_Standard-DHS_2014.cfm?flag=0

## Abbreviations

DHS: Demographic health survey
EDHS: Egyptian Demographic and Health Surveys
