# Supplementary material for: Factors associated with injuries among preschool children in Egypt: demographic and health survey results, 2014
Source: BMC Public Health. 2020 May 1;20:595. doi: 10.1186/s12889-020-08658-w (PMC7193349; doi:10.1186/s12889-020-08658-w)
Supplement: Supplementary file 1 — Additional file 1. Demographic variables: Place of residency. [file 12889_2020_8658_MOESM1_ESM.docx]

**Appendix A:**

**Demographic variables:**

**Place of residency**.

Egypt is divided into 27 governorates, six major subdivisions; five of them are the frontier governorates which are South and North Sini, South Valley, The Red Sea, and Matroh. There are four urban governorates with no rural areas (Cairo, Alexandria, Port Said, and Suez Canal) There are nine governorates which located in the delta region (lower Egypt) and divided into Lower urban and lower rural areas. Another nine governorates which located in the Nile Valley (Upper Egypt) and are divided into Upper Urban and Upper rural areas.

**Wealth index:**

The information collected about the household characteristics used to create a wealth index which reflects the long-term standard of living **(Ruststein and Johnson 2004)**. The three steps procedure used to measure the Wealth index in EDHS 2014 considered the urban-rural differences in household characteristics and asset measures **(Ruststein 2008)**

Rutstein, S.O., and K. Johnson. 2004. The DHS Wealth Index. DHS Comparative Reports No. 6. Calverton, Maryland, USA: ORC Macro.

Rutstein, S. 2008. The DHS Wealth Index: Approaches for Rural and Urban Areas. DHS Working Papers. Calverton, Maryland: Macro International.

The wealth index was divided into the DHS survey into five levels, lowest, second, middle, higher, highest. The wealth was reclassified into three categories by adding the lowest and second level together and the higher and highest level together.

**Educational level:**

The compulsory education in Egypt starts at age of six. There are 6 years of primary education followed by another 6 years of secondary education. The primary 6 years and the first 3 years of secondary education are compulsory.

The educational level was categorized in the DHS questionnaire into five groups No education, incomplete primary, complete primary, secondary education, and higher education. For simplification, the five groups were categorized into three groups by adding incomplete primary and complete primary education together and adding the secondary and higher education group together

**Working status of the mother:**

Informal working is common, and, many times, it is not perceived by women themselves as a work. The questionnaire used Several questions to ensure the working status of the mothers in both the formal and informal sectors

Currently employed is defined as having done work in the past seven days, including persons who did not work in the past seven days but who are regularly employed and were absent from work for leave, illness, vacation, or any other reason.

**Variables used to measure the level of care given to the child:**

The survey used two indicators to measure the level of care given for under-five children. The two indicators were in the form of two questions. 1- Sometimes adults taking care of children have to leave the house to go shopping, wash clothes, or for other reasons and have to leave young children. On how many days in the past week, was (NAME) left alone for more than one? 2- On how many days in the past week, was (NAME) left in the care of another child, that is, someone less than 10 years old, for more than one
