# Supplementary material for: Factors associated with injuries among preschool children in Egypt: demographic and health survey results, 2014
Source: BMC Public Health. 2020 May 1;20:595. doi: 10.1186/s12889-020-08658-w (PMC7193349; doi:10.1186/s12889-020-08658-w)
Supplement: Supplementary file 2 — Additional file 2. The questions used to assess if the child was involved in an injury or an accident: 034 Has (NAME) ever been injured or involved in an accident at home? YES, NO. [file 12889_2020_8658_MOESM2_ESM.docx]

**Appendix B:**

**The questions used to assess if the child was involved in an injury or an accident:**

**034 Has (NAME) ever been injured or involved in an accident at home? YES, NO**

Burn

Fracture

Open wound

Electric shock

Other

**036 Did the injury or accident that (NAME) had at home require medical care? YES, NO**
